# Assessing a single-cell multi-omic analytic platform to characterize *ex vivo*-engineered T-cell therapy products

**DOI:** 10.3389/fbioe.2024.1417070

**Published:** 2024-08-20

**Authors:** Maryam Moshref, Jerry Hung-Hao Lo, Andrew McKay, Julien Camperi, Joseph Schroer, Norikiyo Ueno, Shu Wang, Saurabh Gulati, Somayeh Tarighat, Steffen Durinck, Ho Young Lee, Dayue Chen

**Affiliations:** ^1^ Cell Therapy Engineering and Development, Genentech, South San Francisco, CA, United States; ^2^ Oncology Bioinformatics, Genentech, South San Francisco, CA, United States; ^3^ Pharma Technical Development Bioinformatics, Genentech, South San Francisco, CA, United States; ^4^ Cell and Gene Therapy Business Unit, Mission Bio, South San Francisco, CA, United States; ^5^ Bioinformatics Department, Mission Bio, South San Francisco, CA, United States

**Keywords:** single-cell, multi-omics, engineered CD8^+^ T cells, clustered regularly interspaced short palindromic repeats, heterogeneity, final cell product

## Abstract

Genetically engineered CD8^+^ T cells are being explored for the treatment of various cancers. Analytical characterization represents a major challenge in the development of genetically engineered cell therapies, especially assessing the potential off-target editing and product heterogeneity. As conventional sequencing techniques only provide information at the bulk level, they are unable to detect off-target CRISPR translocation or editing events occurring in minor cell subpopulations. In this study, we report the analytical development of a single-cell multi-omics DNA and protein assay to characterize genetically engineered cell products for safety and genotoxicity assessment. We were able to quantify on-target edits, off-target events, and potential translocations at the targeting loci with per-cell granularity, providing important characterization data of the final cell product. Conclusion: A single-cell multi-omics approach provides the resolution required to understand the composition of cellular products and identify critical quality attributes (CQAs).

## Introduction

T cells play a critical role in protecting the human body from malignant cells through specific recognition by T-cell receptors (TCRs). Cancer immunotherapy augments the T-cell antitumor response, helping the immune system recognize and attack cancer cells (Zeng et al., 2021). Infusion of *ex vivo* modified autologous CD8^+^ T cells specific for the tumor antigens represents an appealing approach for cancer treatment (Manfredi et al., 2020; Wang et al., 2021). TCR α and β chains comprise N-terminal variable and C-terminal constant regions. Targeting TCRs has shown remarkable effectiveness in treating some hematological malignancies (Li et al., 2019). For example, approaches targeting the TCR constant region are practical for developing a shared strategy for all the patients in a clinical trial (Shafer et al., 2022).

CRISPR (clustered regularly interspaced short palindromic repeats) and CRISPR-associated protein (Cas) technology have revolutionized the genome-editing systems from the bench to cell therapy (Mendelsohn and Powis, 2008). In this approach, gRNAs target the desired loci using the PAM sequence, and Cas9 acts on the targeted loci (Wu et al., 2014). There is a possibility that Cas9 also acts on off-target sites in the genome, which may lead to adverse outcomes (Guo et al., 2023). This editing process results in a heterogeneously edited cell population.

One of the major challenges in cell therapy development is the need to ensure the efficacy and safety of the products. Cell-based therapeutics are inherently complex due to their “living drug” nature. Structural variants and off-target edits could potentially affect product safety and efficacy. Therefore, it is critical to implement analytical tools and processes to characterize and test cell and gene therapy (CGT) products. DNA sequencing has revolutionized our understanding of genetic information in the field of medicine, driving significant advancements. However, analyzing complex structural variations and repetitive DNA sequences in human genomes has been challenging by using traditional short-read sequencing methods. To address this, long-read sequencing (LRS) has emerged as a solution, enabling the sequencing of larger DNA fragments ranging from tens to hundreds of kilobase pairs. LRS utilizes innovative techniques like real-time sequencing by synthesis and nanopore-based direct electronic sequencing (Metzker, 2010; Goodwin et al., 2016; Warburton and Sebra, 2023; Szakállas et al., 2024). Despite these advances, conventional bulk analysis in DNA sequencing only provides population-level metrics, which may mask cell-to-cell variation (Yu et al., 2021) and functional differences among cell subpopulations. This limitation highlights the need for more comprehensive characterization of cell products (Miles et al., 2020). Single-cell DNA and cell-surface protein sequencing offers a solution by deconvoluting the heterogeneity of engineered cells, allowing simultaneous assessment of the genotype and phenotype. This in-depth analysis provides insights into DNA edits and their potential association with different subpopulations, facilitated by measuring predefined cell surface markers. Such a multi-omics approach enables product characterization at a precise level of granularity, thus identifying product critical quality attributes (CQAs). The Tapestri technology is an amplicon-based single-cell DNA sequencing platform and is currently the only system capable of simultaneously providing genotype and phenotype data from the same cell (Demaree et al., 2021; ten et al., 2020). Here, we modified CD8^+^ T cells by disrupting the constant region of T-cell receptor alpha constant (TRAC) and T-cell receptor beta constant 1 and 2 (TRBC1 and TRBC2) (Oh et al., 2019; Oh et al., 2022) to assess genotypic heterogeneity and characterize the CRISPR–Cas9 engineered cell products at the single-cell level using the Tapestri platform.

## Materials and methods

### Single-cell DNA sequencing and protein library preparation

Single-cell, amplicon-based DNA and protein sequencing were performed on edited T cells from four different donors (edited CGT products) upon simultaneous knockout of the three loci: TRAC, TRBC1, and TRBC2. We analyzed the 40 custom-designed amplicons to investigate the off-target and translocation events in common predicted regions using the Tapestri (v.2) platform (Mission Bio) (Demaree et al., 2021; ten et al., 2020). We used donor-matched unedited cells as the control. Fourteen TotalSeq oligo-conjugated antibodies from BioLegend were used for cell-surface protein analysis (Supplementary Table S2). The assay was performed according to the manufacturer’s (Mission Bio) standard protocol (Ediriwickrema et al., 2020; Miles et al., 2020; Dillon et al., 2021; Ho et al., 2024). Cryopreserved CGT products were thawed using 37°C PRIME-XV media, followed by washing three times with pre-warmed PBS at 37°C. Cell pellets were resuspended in Cell Staining Buffer (CSB, BioLegend, no. 420201) at 25,000 cells per μL in a total volume of 40 μL. The cell suspension was blocked using TruStain FcX (BioLegend) and Blocking Buffer (Mission Bio) for 15 min on ice and then stained with a pool of 14 oligo-conjugated antibodies for 30 min on ice, followed by washing multiple times with the CSB according to the Tapestri Single-Cell DNA + Protein Sequencing User Guide. Cells were counted and resuspended in Cell Buffer (Mission Bio) at 4,000 cells per μL and deposited on a Tapestri microfluidics cartridge. Briefly, single cells were encapsulated in the encapsulation oil (Mission Bio) with Lysis Buffer (Mission Bio) to create a cell emulsion. The cell-identifying barcode was then integrated into each amplicon as part of a triple-primer PCR system. DNA PCR products were then isolated and purified with AMPure XP beads (Beckman Coulter), and the supernatants were retained to capture the protein library. Two microliters (5 μM) biotin oligo (Mission Bio) was added to the protein PCR product at 96°C for 5 min, followed by incubation on ice for 5 min. The protein PCR product is then extracted using streptavidin beads (Mission Bio), followed by adding protein indices. Both DNA and protein libraries were purified by AMPure XP beads, and the final concentration was measured by using a Qubit (1x high-sensitivity dsDNA kit, Invitrogen). All libraries were quantified by Agilent 2100 Bioanalyzer (DNA 1000 kit—Agilent Technologies).

### Sequencing

DNA and protein libraries were sequenced 2 × 150 bp (paired-end) for 20 and 70 million reads, respectively, on Illumina NovaSeq by MedGenome Inc.

### Single-cell DNA sequencing and protein data analysis

A minimum of 40 million paired-end read FASTQ files were processed by Tapestri Genome Editing Pipeline on the Genentech high-performance computing (HPC). Paired-end reads were trimmed by Cutadapt (Martin, 2011; Bolger et al., 2014) to remove sequencing adapters and discard short reads (< 30 bp). Trimmed reads were mapped to the human hg38 reference genome by BWA-MEM, and cell calling was performed by Tapestri Cellfinder to split bam files per identified cell barcode. The cells are genotyped using GATK (McKenna et al., 2010) with a joint calling approach that follows GATK Best Practices recommendations (Depristo et al., 2011; Van der Auwera et al., 2013). Each cell and amplicon were haplotypes, and the edited loci were annotated with on-/off-target information based on CRISPR gRNA design. Variants were filtered if they were present in > 80% cells to remove potential germline variants. Cells and amplicons were removed if combination reads were less than eight reads. Filtered cells were used to quantify the number/percentage of cells with insertion–deletion. For translocations, paired-end reads that aligned to different chromosomes (chimeric reads) were identified by Tapestri AssignPrimers, and reads lacking a primer were removed. The chimeric reads were used to discover the translocation breakpoint and quantify the number/percentage of cells with translocation events. Protein data were analyzed by Tapestri Protein Pipeline v2.0.1 on the Genentech HPC. For the downstream analysis and visualization of protein data, h5 files were read into Mosaic, and the sequenced cells were subsets based on called cells from DNA data. The protein data were log-normalized, centered, and scaled on a per sample basis, and the top four principal components were used as the input in the KNN neighbor identification. Subsequent community identification was performed with the “FindClusters” function with a resolution variable of 0.25. The clone information was applied as metadata, and all subsequent analyses of ridge plots, feature heatmaps, and UMAPs were performed on Mosaic.

### Frameshift analysis

Frameshift analysis was performed on a per-cell basis for the three on-target sites and one off-target site for each donor. The editing status was assessed as follows:

Cells without enough genotyping information were determined to be “no-calls.” Cells where both alleles were genotyped without any indels were determined to be wild type. The cells with bi-allelic frameshift (FS) contained alleles with an indel, and the indel lengths for both were not a multiple of 3, whereas the cells in which both alleles had an indel and the indel lengths were a multiple of 3 were considered bi-allelic non-frameshift (NFS). Mono-allelic NFS were cells that contained only one edited allele, in which the length of the indel was a multiple of 3, while mono-allelic FS covered two cases: either both alleles were edited, but one of them was a multiple of 3 and the other allele was not a multiple of 3, or only one allele was edited and it was not a multiple of 3.

### Statistical analysis

The Python package Seaborn was used for plotting graphs. Off-target activity for Off Target 2 (OT2) with sample information was plotted using the Python package Seaborn. Significance was assessed using the Python package SciPy, specifically the statistic nonparametric unpaired Wilcoxon rank-sum test. Results were reported as significant if their *p*-value passed the 0.05 threshold.

### Primary human CD8^+^ T-cell isolation and culture

Blood from four healthy donors was purchased from CGT Global. CD8^+^ T cells were isolated by positive selection using the StraightFrom Buffy Coat CD8 MicroBead Kit according to the manufacturer’s instructions (Miltenyi Biotec), and cells were cultured at 1 million cells/mL in PRIME-XV T-cell CDM media (Irvine Scientific) and supplemented with 25 ng/mL of IL-7 and 50 ng/mL of IL-15 (Miltenyi Biotec). CD8^+^ cells were activated using 1:100 dilution of T-cell TransAct (Miltenyi Biotec) and cultured for 48 h before electroporation.

### gRNA and RNP assemblies

All sgRNA sequences were ordered from Integrated DNA Technologies (IDT).

The TRAC sgRNA sequence was ACA​AAA​CTG​TGC​TAG​ACA​TG, and the TRBC sgRNA sequence was TGG​CTC​AAA​CAC​AGC​GAC​CT. The TRAC and TRBC genes were knocked out by forming RNP complexes, as described previously (Camperi et al., 2022; Goyon et al., 2022). Briefly, the sgRNAs were reconstituted in Nuclease-free Duplex Buffer (IDT) to obtain a 200 µM stock solution. Each sgRNA and recombinant Cas9 (SpyFi^TM^; Aldevron) were mixed at an sgRNA: Cas9 ratio of 3:1 and incubated at room temperature for 15 min. Cas9–RNPs were assembled separately and then mixed using equal volumes.

### Nucleofection and cell expansion

Activated CD8^+^ T cells were pelleted, washed with PBS, and gently resuspended in P3 buffer with supplement (Lonza Bioscience) at 2 million cells per 20 µL with sgTRAC and sgTRBC RNPs (knock-out). For a single nucleofection, 60 pmol of combined TRAC and TRBC Cas9–RNPs was used. The mixture was then transferred to a single well of a 16-well 4D-Nucleofector Cuvette (Lonza Bioscience) and pulsed using the EH115 code. Unedited CD8^+^ T cells were used as the control and were added to the Lonza electroporation cuvette but not electroporated. Cells were rested at room temperature for 15 min after electroporation and cultured at 37°C in a 24-well G-Rex plate (Wilson Wolf) in PRIME-XV medium supplemented with 25 ng/mL of IL-7 (Miltenyi) and 50 ng/mL of IL-15 (Miltenyi) for 7 days of expansion. Cells were cryopreserved in CryoStor CS10 (STEMCELL Technologies) and stored in liquid nitrogen until library preparation.

## Results

### Single-cell DNA sequencing detects CRISPR–Cas9-induced TRAC and TRBC gene disruptions

Gene-edited products are heterogeneous and require thorough characterization. Here, we assessed an engineered CD8^+^ T-cell product with three simultaneous gene knockouts, namely, the constant region of the TCRα (TRAC locus) and two constant regions of TCRβ (TRBC1 and TRBC2 loci). To characterize the T-cell products, we single-cell encapsulated, barcoded, and prepared DNA libraries using Mission Bio’s Tapestri platform. Libraries from edited and unedited T cells were generated in parallel from four donors with two replicates for each condition, resulting in a total of 16 individual libraries. All 16 libraries passed the Tapestri assay performance criteria including the total number of the reads, % Q30 score, % reads mapped to the genome, mean reads per cell per amplicon, number of the cells, % reads mapped to the target, and % reads mapped to the cells and panel uniformity (Supplementary Figure S1).

**FIGURE 1:**
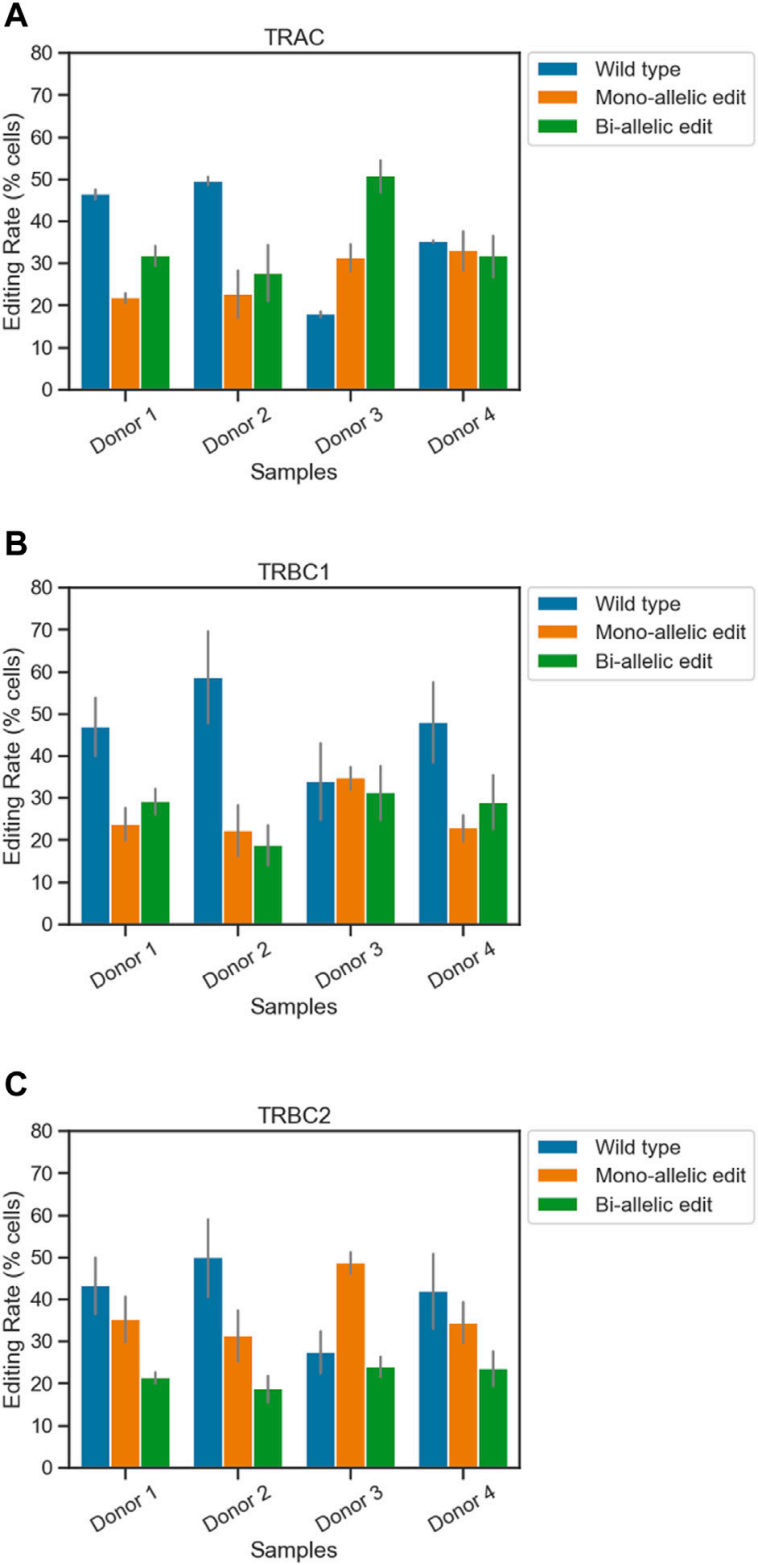
Simultaneous quantification of edits at three loci in engineered T cells using single-cell DNA sequencing. The frequencies of on-target edits at **(A)** TRAC, **(B)** TRBC1, and **(C)** TRBC2 were measured using the Tapestri assay. Bars represent mean ± SEM from two technical duplicate assay runs (Table 1). Each run encapsulated and sequenced at least 4,000 cells.

For the TRAC amplicon, the highest editing rate was observed from donor 3 (82%) and the lowest editing rate was observed from donor 2 (50%). Two out of four samples showed higher bi-allelic edits compared to mono-allelic edits in TRAC (Figure 1A; Table 1). Consistent with TRAC editing rates, TRBC1 and TRBC2 were most frequently edited in donor 3 (65% and 73%) and least frequently edited in donor 2 (40% and 50%) respectively. In contrast to TRAC and TRBC1, TRBC2 exhibited a higher mono-allelic than bi-allelic editing rate (Figures 1B, C; Table 1). Frameshift analysis confirmed that the TRAC, TRBC1, and TRBC2 loci were edited (Supplementary Figure S2). The frequency of frameshift editing was approximately ∼90% for the TRAC gRNA and closer to ∼74% for TRBC1 and ∼50% for the TRBC2 gRNAs.

**TABLE 1:** Frequency of on-target edits at TRAC, TRBC1, and TRBC2. Mean ± SEM from two technical duplicate assay runs.

Locus	Sample	Genotype	Mean	SEM
TRAC	Donor 1	Bi-allelic edit	31.805	1.695
		Mono-allelic edit	21.765	0.885
		Wild type	46.43	0.81
	Donor 2	Bi-allelic edit	27.66	4.72
		Mono-allelic edit	22.725	4.015
		Wild type	49.61	0.7
	Donor 3	Bi-allelic edit	50.74	2.66
		Mono-allelic edit	31.35	2.24
		Wild type	17.91	0.43
	Donor 4	Bi-allelic edit	31.695	3.415
		Mono-allelic edit	33	3.25
		Wild type	35.31	0.16
TRBC1	Donor 1	Bi-allelic edit	29.235	2.135
		Mono-allelic edit	23.81	2.75
		Wild type	46.955	4.885
	Donor 2	Bi-allelic edit	18.885	3.375
		Mono-allelic edit	22.37	4.29
		Wild type	58.735	7.665
	Donor 3	Bi-allelic edit	31.275	4.535
		Mono-allelic edit	34.77	1.89
		Wild type	33.95	6.43
	Donor 4	Bi-allelic edit	29.07	4.49
		Mono-allelic edit	22.845	2.215
		Wild type	48.085	6.705
TRBC2	Donor 1	Bi-allelic edit	21.395	0.945
		Mono-allelic edit	35.255	3.785
		Wild type	43.355	4.735
	Donor 2	Bi-allelic edit	18.75	2.23
		Mono-allelic edit	31.355	4.285
		Wild type	49.895	6.515
	Donor 3	Bi-allelic edit	23.98	1.67
		Mono-allelic edit	48.61	1.86
		Wild type	27.41	3.53
	Donor 4	Bi-allelic edit	23.595	2.855
		Mono-allelic edit	34.44	3.41
		Wild type	41.97	6.26

### Single-cell sequencing quantifies the co-occurrence and zygosity of multiple simultaneous on-target edits.

**FIGURE 2:**
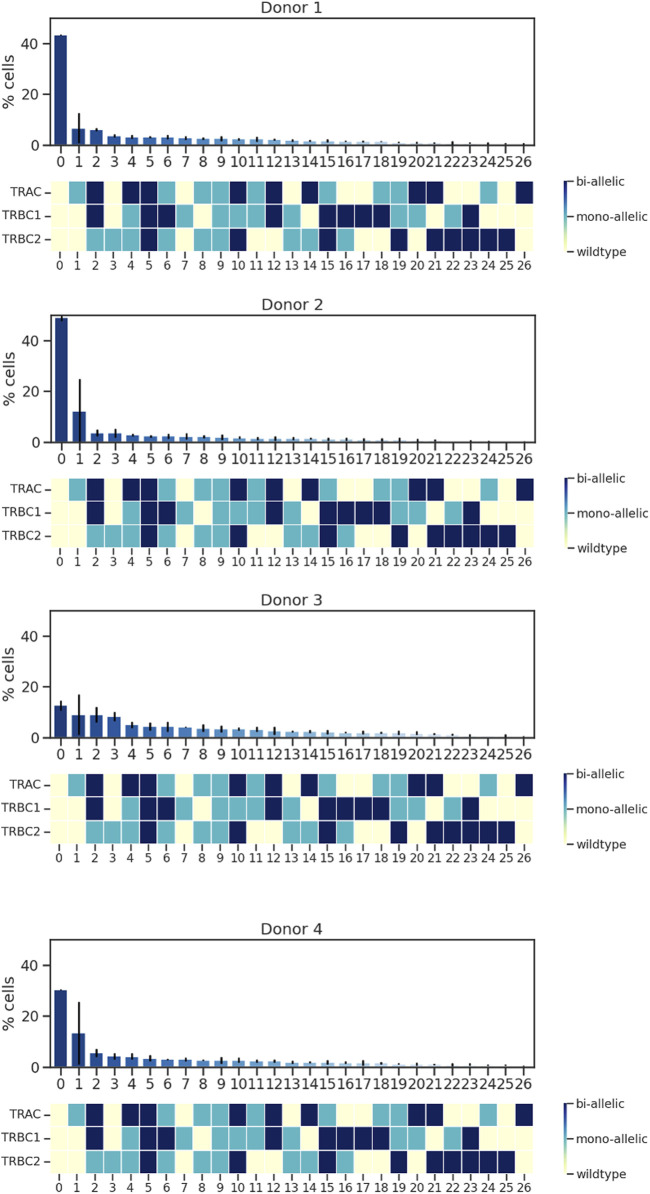
Analysis of TRAC, TRBC1, and TRBC2; editing rate; and edit co-occurrence rate. Data from four donors and two replicates are shown. Bars represent mean ± SD from two technical duplicate assay runs (Supplementary Material S2). To illustrate all the possible combinations, we assigned a color to each state of the edit as follows: yellow was assigned to wild type, light blue to mono-allelic edit, and dark blue to biallelic edit, and then we assigned the % edit related to each combination at the top of the color-coded map. Each run encapsulated and sequenced at least 4,000 cells.

When characterizing the on-target edit of each of the three loci, we must consider that there are multiple genotypic outcomes: wild type, mono-allelically edited, and bi-allelically edited. These outcomes can then co-occur in a single cell in many combinations. For example, if CRISPR–Cas9 edits all three loci of a single cell, mono-allelically or bi-allelically, it generates a triple mono-allelic or bi-allelic edited cell. In some cases, only two loci get edited mono-allelically or bi-allelically or one locus gets edited. A triple wild-type cell is a cell with no edits. Co-occurrences of all these possibilities create different cell populations with various genotypic outcomes. Upon deploying single-cell sequencing using Tapestri, we detected different cell populations with different genotypic outcomes. We found that the rates of triple wild-type edits in the four donors ranged from 48% to 11%, whereas the rates of both triple mono-allelic and bi-allelic edits were much lower, between 2% and 5% (Figure 2).

### Single-cell sequencing quantifies the CRISPR–Cas9 off-target events of multiple simultaneous on-target edits in engineered T cells

**FIGURE 3:**
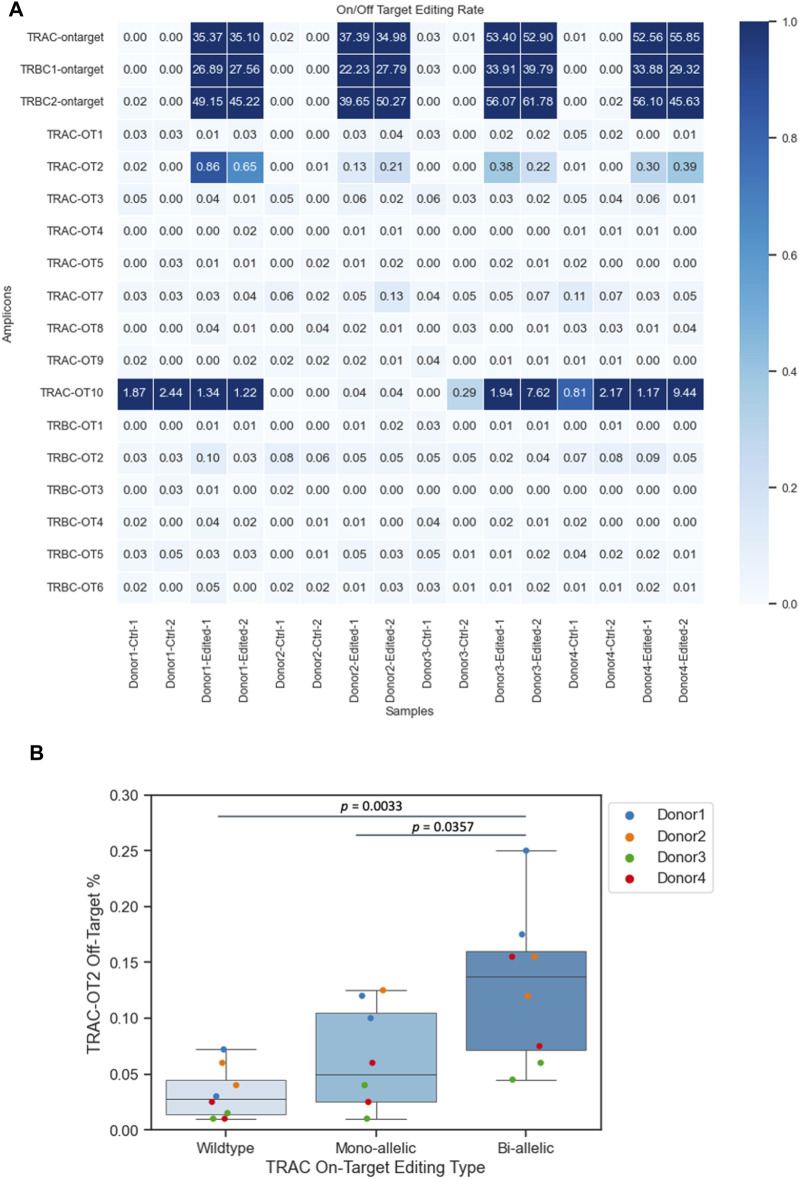
Frequencies of CRISPR–Cas9 off-target events. **(A)** TRAC-OT2 identified as the off-target editing event. **(B)** TRAC-OT2 and TRAC (on target) co-occurrence (*p*-value passed the 0.05 threshold). The percentage of TRAC-OT2 increased when TRAC on target edits increased on average for wild type (0.03275 ± 0.00818), mono-allelic (0.063125 ± 0.0162), and bi-allelic (0.129375 ± 0.0242). Data represented as mean ± SEM from four donors and two technical duplicates from each donor from edited and unedited controls.

To identify nonspecific genetic modifications, we assessed the TRAC off-target (TRAC-OT) editing rates at nine predicted sites and TRBC off-target (TRBC-OT) editing rates at five sites (Roth et al., 2018; Oh et al., 2022) (Figure 3A). We compared the on-target insertion or deletion (INDEL) rates (shown at the top of the heatmap) to the indel rates at TRAC-OT sites. TRAC-OT2 in edited samples exhibited a higher indel rate when compared to unedited samples across the donors, and it was identified as an off-target event, which was also confirmed by frameshift analysis (Supplementary Figure S3). The TRAC-OT2 frequency of frameshift editing across the four donors was between ∼22% and ∼67%. For TRAC-OT10 off-target rates, three donors out of four (donors 1, 2, and 4) had genomic background control samples containing edits at this site; therefore, TRAC-OT10 could not be considered an off-target editing event (Figure 3A). Furthermore, most cells for TRAC-OT10 were no calls (not enough read depth), meaning this amplicon was a low-performing amplicon and inherently noisy (Supplementary Figure S4). The data suggest that the percentage of cells with edits in TRAC-OT2 increased as on-target editing at the TRAC site increased. Cells with bi-allelic editing at the TRAC site were more likely to be edited at the TRAC-OT2 site compared to cells with mono-allelic edits or no edit at the TRAC site (Figure 3B).

### Single-cell sequencing quantifies the chromosomal translocations and chromosomal breakpoints of multiple simultaneous on-target edits in engineered T cells

**FIGURE 4:**
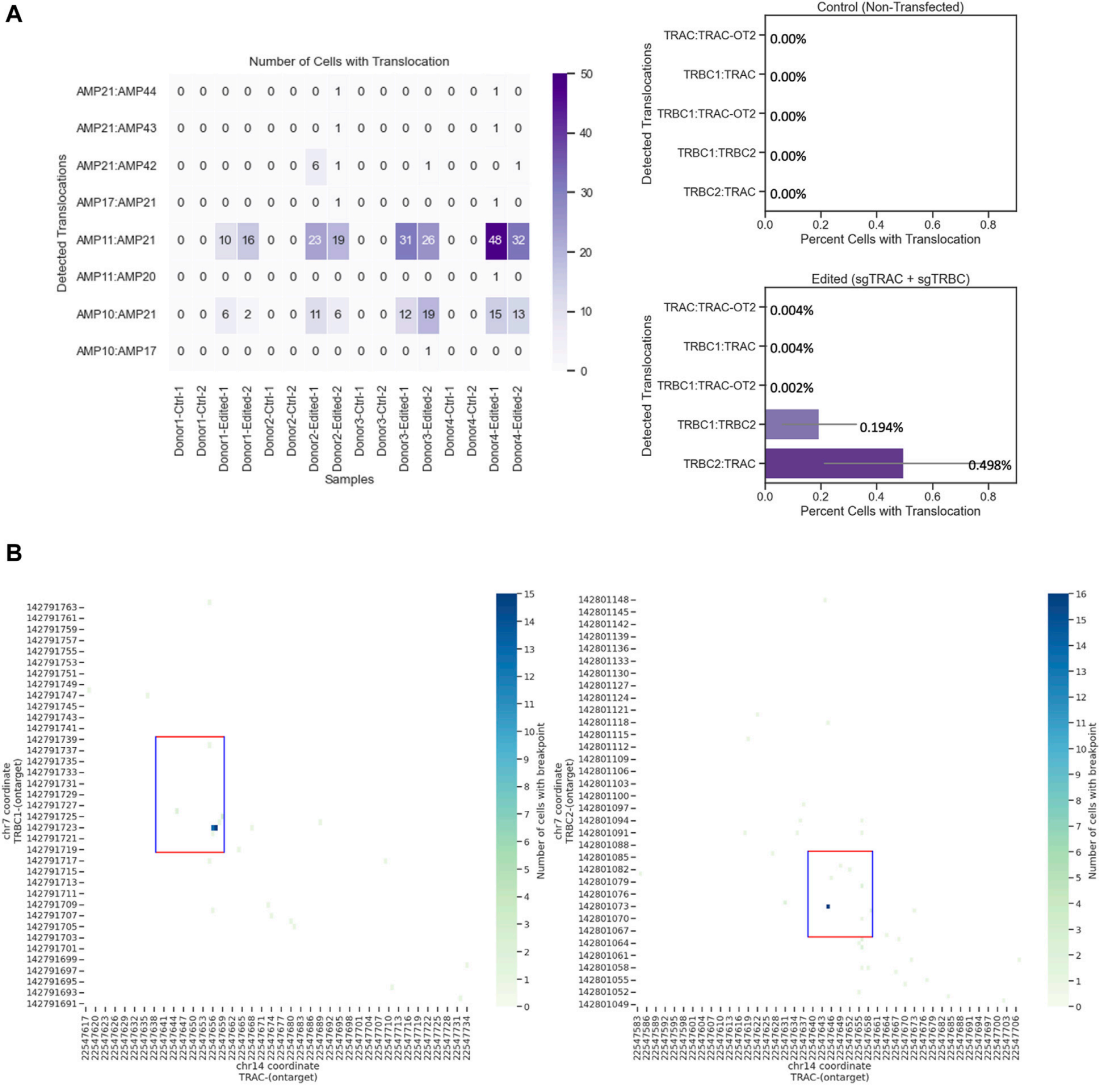
Chromosomal translocations and chromosomal breaking point in engineered T cells at the single-cell level. **(A)** TRBC2-TRAC (Amp11 and 21, respectively) is the most commonly identified translocation. Left: number of cells with translocation from edited and unedited controls from four donors and two replicates. Right: quantification of cells with translocation. Data show the mean values with error bars. **(B)** Chromosomal breakpoints are within the 20-bp editing window. Each run encapsulated and sequenced at least 4,000 cells.

Editing three loci creates six different quantifiable translocations (Oh et al., 2022) (Supplementary Table S1). We identified four of these translocations between the TRAC and TRBC1 loci and the TRAC and TRBC2 loci. We could not design amplicons within the 270-bp size range, which is the approximate amplicon size for the Tapestri platform, to detect any possible translocations between TRBC1 and TRBC2 due to the similarity of these two regions (Maciocia et al., 2017). The most detected translocation was between AMP11 (TRBC2) and AMP21 (TRAC) (Figure 4A), which was confirmed with orthogonal ddPCR data (Supplementary Figure S5). AMP21 and AMP11 amplicons began and ended at chr14: 22547535–22547765 and chr7: 142800983–142801198, respectively (Supplementary Material S1). We also observed translocations between AMP21 (TRAC) and AMP42 (TRBC2), AMP10 (TRBC1) and AMP21 (TRAC), AMP21 (TRAC) and AMP43 (TRBC1), and AMP21 (TRAC) and AMP44 (TRBC2). No translocations were detected in unedited samples (Figure 4A). Assessing the translocation events at the single-cell level revealed further details on structural variants and zygosity for each allele (Figure 4B). It was determined that most identified breakpoints were within the 20-bp editing windows, indicating that these translocations were at least in part facilitated by CRISPR editing (Figure 4B).

### Phenotypic profiles are similar across different editing combinations (zygosity status) of TRAC and TRBC genes.

Furthermore, we utilized Tapestri assays linking the genotypic attributes to the immunophenotype of our cell therapy product. We designed an antibody panel consisting of 14 TotalSeq-D antibodies to detect cells with the following phenotypes: naïve (CD45RA + CD45RO− CD27 + CD95−), stem cell memory (CD45RA + CD45RO− CD27 + CD95+), terminal effector (CD45RA + CD45RO−CD27− CD95+), central memory (CD45RA+/− CD45RO + CD27^+^), and effector memory (CD45RA+/− CD45RO + CD27−). First, we determined the % of different subpopulations of engineered CD8 T cells consisting of 0.0% naïve, 25.0% T memory stem (TSCM) cells, 0.8% terminal effector (TE), 54.0% central memory (CM), and 13.0% effector memory (EM) cells, which were almost comparable with 0.0% naïve, 25.5% T memory stem (TSCM) cells, 5.3% terminal effector (TE), 55.2% central memory (CM), and 13.6% effector memory (EM) cells in flow cytometry data (Supplementary Figure S6).

**FIGURE 5:**
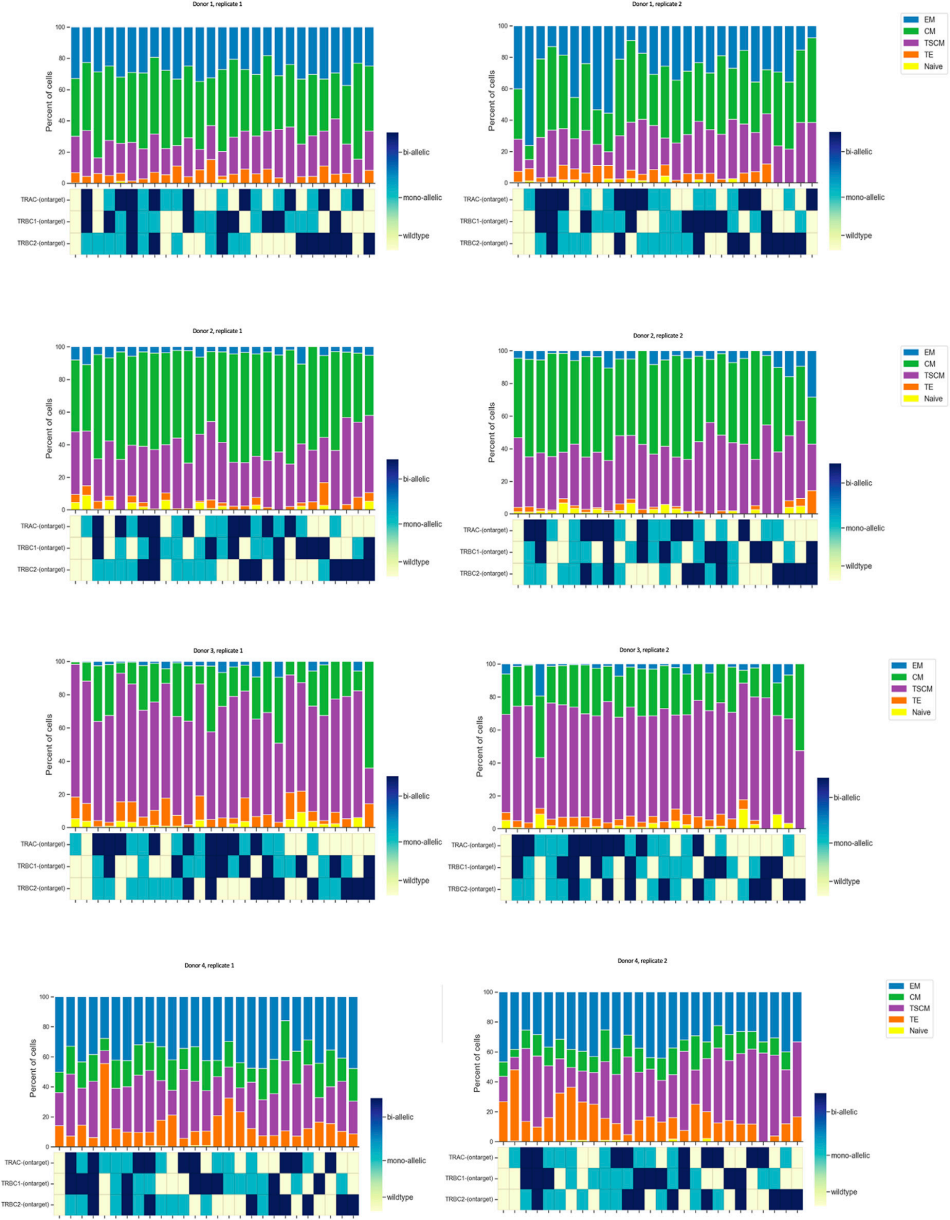
Quantified distribution of protein populations based on DNA editing status across different donors. In order to illustrate all possible combinations, we assigned a color to each state of the edit as follows: color red was assigned to wild type, blue to mono-allelic edit, and green to bi-allelic edit. We plotted this as a stacked bar graph of the % subpopulation (each color coded differently) of CD8 T cells for each edit combination at the top of the map. Data are from four donors, and two technical replicates are shown side by side. Each run encapsulated and sequenced at least 4,000 cells.

We plotted the % of protein subpopulation distribution within each on-target editing combination. Between 5% and 0.4% of the cells in certain markers did not show bimodal expression; therefore, cells with “ambiguous” protein expression as well as cells with not enough reads in DNA data were removed from the analysis. As illustrated in Figure 2, there are many combinations (wild type, mono-allelic, and bi-allelic) in the DNA profile of CGT products when assessing the on-target edit co-occurrence of the three loci. We then conducted scDNA-seq and simultaneously profiled DNA and cell-surface proteins in edited and unedited samples from four healthy donors in duplicates. Although TSCM and CM populations have more DNA edits compared to the TE and EM, no specific trend was observed with regards to editing status and protein identified cell types (Figure 5). The sample number did not allow us to perform any statistical analysis. We showed that the CRISPR editing system is not predisposed to target a particular CD8 T-cell subset comparing the triple wild-type events with unedited samples (Supplementary Figure S7). This approach to single-cell multi-omics analysis confirmed that mono- and bi-allelic edits made at all three loci were randomly distributed across all CD8 T cells, without bias or over-representation in any specific subpopulation.

## Discussion

By applying Mission Bio’s Tapestri single-cell DNA-seq assay, we assessed the feasibility and performance of the platform. Our study uncovered the landscape of the CGT product architecture at single-cell resolution, and simultaneous DNA and protein profiling enabled genotype-to-phenotype correlation at single-cell resolution. This study revealed novel information, such as on-target and off-target edit co-occurrences, biallelic and mono-allelic translocations, and chromosomal breakpoints.

The ability of bulk sequencing data to portray the heterogeneity of CGT products is inherently limited (Wienert et al., 2019; Li et al., 2021; Yu et al., 2021). Conventional bulk characterization methods have resolution limitations that lead to the need for multiple instruments and platforms to enable a comprehensive characterization. The data output from each platform must be integrated to gain meaningful insights, a challenge that cannot be directly addressed when inferring correlations across multiple assays. Although bulk analysis is inexpensive, faster, and good practice (GxP)-compliant, CGT samples are often limited and may not be available to be tested on multiple platforms. In addition, current analytical methods for genotype measurements are based on bulk analysis, which does not really resolve the intrinsic heterogeneity among the gene-edited cell populations (Navin, 2015; Stuart and Satija, 2019; Li et al., 2021; Yu et al., 2021). Although other conventional scDNA-seq methods can address some of these challenges, they often require lengthy processes for single-cell isolation and long turnaround time as well as whole-genome amplification, which could introduce technical artifacts (Wang and Navin, 2015; Morita et al., 2021). Tapestri uses scDNA-seq approaches that combine droplet microfluidics and molecular barcoding to enable a high-throughput targeted amplicon generation to delineate the genetic heterogeneity produced by CRISPR–Cas9 alterations (Pellegrino et al., 2018). In this study, we have used Tapestri to simultaneously profile DNA edits and cell surface protein markers at the single-cell level. The analysis has advanced our understanding of how editing cells at the DNA level may not necessarily result in corresponding changes phenotypically, thus enabling a deeper understanding of the cellular products. We investigated 40 amplicons for off-target, on-target, edit co-occurrence, and translocation events with a remarkable level of complexity in the simultaneous knocking out of three loci, namely, TRAC, TRBC1, and TRBC2. Our study achieved two key findings. First, we comprehensively evaluated CRISPR editing efficiency, measuring both the desired triple edits and off-target effects. Second, we analyzed single-cell editing outcomes, considering both the number of edited alleles (zygosity) and co-occurrences of edits across all three targeted loci. This analysis revealed no preferential targeting of specific CD8 T-cell subsets by the CRISPR system. In simpler terms, editing affected the entire T-cell population, not just a specific subpopulation identified by cell-surface proteins. We used cell-surface proteins to identify T-cell subpopulations, but this analysis solely served to demonstrate that editing was not specific to a particular subpopulation. As the targeted genes (TRAC and TRBC) lack connection to cell-surface proteins, we do not anticipate changes in their expression. Most importantly, this study uncovered four putative translocations across the on-target loci, which is a direct safety attribute. In conclusion, the single-cell multi-omics approach provides the resolution required to understand the composition of cellular products and CQAs. Overall, these are all important parameters for genomic integrity and are applicable both in the world of platform development to optimize editing outcomes as well as during manufacturing, where product consistency must be assessed prior to introduction into patients. It is noteworthy that this is still an estimation of the true extent of heterogeneity. Future techniques that accommodate higher numbers of cells used on the platform, more comprehensive coverage of the genome, and larger amplicons (long read sequencing) will further shed light on the complexity of the characterization of CGT products. Future evaluation of edited T cells collected from clinical development programs could create a prospective plan to deepen the information about predictive and prognostic outcomes of CGT products.

## Data Availability

Data deposited at: Gene Expression Omnibus (GEO) (https://www.ncbi.nlm.nih.gov/geo/). The accession is GSE274350.

## References

[B1] Bolger A. M. Lohse M. Usadel B. (2014). Trimmomatic: a flexible trimmer for Illumina sequence data. Bioinformatics 30 (15), 2114–2120. 10.1093/bioinformatics/btu170 24695404 PMC4103590

[B2] Camperi J. Moshref M. Dai L. Lee H. Y. (2022). Physicochemical and functional characterization of differential CRISPR-cas9 ribonucleoprotein complexes. Anal. Chem. 94 (2), 1432–1440. 10.1021/acs.analchem.1c04795 34958212

[B3] Demaree B. Delley C. L. Vasudevan H. N. Peretz C. A. C. Ruff D. Smith C. C. (2021). Joint profiling of DNA and proteins in single cells to dissect genotype-phenotype associations in leukemia. Nat. Commun. 12 (1), 1583. 10.1038/s41467-021-21810-3 33707421 PMC7952600

[B4] Depristo M. A. Banks E. Poplin R. Garimella K. V. Maguire J. R. Hartl C. (2011). A framework for variation discovery and genotyping using next-generation DNA sequencing data. Nat. Genet. 43 (5), 491–498. 10.1038/ng.806 21478889 PMC3083463

[B5] Dillon L. W. Ghannam J. Nosiri C. Gui G. Goswami M. Calvo K. R. (2021). Personalized single-cell proteogenomics to distinguish acute myeloid leukemia from non-malignant clonal hematopoiesis. Blood Cancer Discov. 2 (4), 319–325. 10.1158/2643-3230.bcd-21-0046 34258102 PMC8265308

[B6] Ediriwickrema A. Aleshin A. Reiter J. G. Corces M. R. Kohnke T. Stafford M. (2020). Single-cell mutational profiling enhances the clinical evaluation of AML MRD. Blood Adv. 4 (5), 943–952. 10.1182/bloodadvances.2019001181 32150611 PMC7065471

[B7] Goodwin S. McPherson J. D. McCombie W. R. (2016). Coming of age: ten years of next-generation sequencing technologies. Nat. Rev. Genet. 17 (6), 333–351. 10.1038/nrg.2016.49 27184599 PMC10373632

[B8] Goyon A. Nguyen D. Boulanouar S. Yehl P. Zhang K. (2022). Characterization of impurities in therapeutic RNAs at the single nucleotide level. Anal. Chem. 94 (48), 16960–16966. 10.1021/acs.analchem.2c04681 36410036

[B9] Guo C. Ma X. Gao F. Guo Y. (2023). Off-target effects in CRISPR/Cas9 gene editing. Front. Bioeng. Biotechnol. 11, 1143157. 10.3389/fbioe.2023.1143157 36970624 PMC10034092

[B10] Ho I. L. Li C. Y. Wang F. Zhao L. Liu J. Yen E. Y. (2024). Clonal dominance defines metastatic dissemination in pancreatic cancer. Sci. Adv. 10 (11), eadd9342. 10.1126/sciadv.add9342 38478609 PMC12697574

[B11] Li D. Li X. Zhou W. L. Huang Y. Liang X. Jiang L. (2019). Genetically engineered T cells for cancer immunotherapy. Signal Transduct. Target. Ther. 4 (1), 35–17. 10.1038/s41392-019-0070-9 31637014 PMC6799837

[B12] Li Y. Ma L. Wu D. Chen G. (2021). Advances in bulk and single-cell multi-omics approaches for systems biology and precision medicine. Brief. Bioinform 22 (5), bbab024–18. 10.1093/bib/bbab024 33778867

[B13] Maciocia P. M. Wawrzyniecka P. A. Philip B. Ricciardelli I. Akarca A. U. Onuoha S. C. (2017). Targeting the T cell receptor β-chain constant region for immunotherapy of T cell malignancies. Nat. Med. 23 (12), 1416–1423. 10.1038/nm.4444 29131157

[B14] Manfredi F. Cianciotti B. C. Potenza A. Tassi E. Noviello M. Biondi A. (2020). TCR redirected T cells for cancer treatment: achievements, hurdles, and goals. Front. Immunol. 11, 1689. 10.3389/fimmu.2020.01689 33013822 PMC7494743

[B15] Martin M. (2011). Cutadapt removes adapter sequences from high-throughput sequencing reads. EMBnet J. 17 (1), 10–12. 10.14806/ej.17.1.200

[B16] McKenna A. Hanna M. Banks E. Sivachenko A. Cibulskis K. Kernytsky A. (2010). The Genome Analysis Toolkit: a MapReduce framework for analyzing next-generation DNA sequencing data. Genome Res. 20 (9), 1297–1303. 10.1101/gr.107524.110 20644199 PMC2928508

[B17] Mendelsohn J. Powis G. (2008). From bench to bedside with targeted therapies. Biochim. Biophys. Acta Mol. Basis Dis., 521–530. 10.1016/b978-141603703-3.10043-3

[B18] Metzker M. L. (2010). Sequencing technologies — the next generation. Nat. Rev. Genet. 11 (1), 31–46. 10.1038/nrg2626 19997069

[B19] Miles L. A. Bowman R. L. Merlinsky T. R. Csete I. S. Ooi A. T. Durruthy-Durruthy R. (2020). Single-cell mutation analysis of clonal evolution in myeloid malignancies. Nature 587 (7834), 477–482. 10.1038/s41586-020-2864-x 33116311 PMC7677169

[B20] Morita K. Wang F. Jahn K. Hu T. Tanaka T. Sasaki Y. (2021). Author Correction: clonal evolution of acute myeloid leukemia revealed by high-throughput single-cell genomics. Nat. Commun. 12 (1), 2823. 10.1038/s41467-021-23280-z 33972555 PMC8110810

[B21] Navin N. E. (2015). Delineating cancer evolution with single cell sequencing. Sci. Transl. Med. 7 (296), 296fs29. 10.1126/scitranslmed.aac8319 PMC4785805 26180099

[B22] Oh S. A. Seki A. Rutz S. (2019). Ribonucleoprotein transfection for CRISPR/Cas9-Mediated gene knockout in primary T cells. Curr. Protoc. Immunol. 124 (1), e69. 10.1002/cpim.69 30334617

[B23] Oh S. A. Senger K. Madireddi S. Akhmetzyanova I. Ishizuka I. E. Tarighat S. (2022). High-efficiency nonviral CRISPR/Cas9-mediated gene editing of human T cells using plasmid donor DNA. J. Exp. Med. 219 (5), e20211530. 10.1084/jem.20211530 35452075 PMC9040063

[B24] Pellegrino M. Sciambi A. Treusch S. Durruthy-Durruthy R. Gokhale K. Jacob J. (2018). High-throughput single-cell DNA sequencing of acute myeloid leukemia tumors with droplet microfluidics. Genome Res. 28 (9), 1345–1352. 10.1101/gr.232272.117 30087104 PMC6120635

[B25] Roth T. L. Puig-Saus C. Yu R. Shifrut E. Carnevale J. Li P. J. (2018). Reprogramming human T cell function and specificity with non-viral genome targeting. Nature 559 (7714), 405–409. 10.1038/s41586-018-0326-5 29995861 PMC6239417

[B26] Shafer P. Kelly L. M. Hoyos V. (2022). Cancer therapy with TCR-engineered T cells: current strategies, challenges, and prospects. Front. Immunol. 13, 835762. 10.3389/fimmu.2022.835762 35309357 PMC8928448

[B27] Stuart T. Satija R. (2019). Integrative single-cell analysis. Nat. Rev. Genet. 20 (5), 257–272. 10.1038/s41576-019-0093-7 30696980

[B28] Szakállas N. Barták B. K. Valcz G. Nagy Z. B. Takács I. Molnár B. (2024). Can long-read sequencing tackle the barriers, which the next-generation could not? A review. Pathology Oncol. Res. 30, 1611676. 10.3389/pore.2024.1611676 PMC11137202 38818014

[B29] ten Hacken E. Clement K. Li S. Hernández-Sánchez M. Redd R. Wang S. (2020). High throughput single-cell detection of multiplex CRISPR-edited gene modifications. Genome Biol. 21 (1), 266–311. 10.1186/s13059-020-02174-1 33081820 PMC7574538

[B30] Van der Auwera G. A. Carneiro M. O. Hartl C. Poplin R. del Angel G. Levy-Moonshine A. (2013). From FastQ data to high-confidence variant calls: the genome analysis toolkit best practices pipeline. Curr. Protoc. Bioinforma. 43 (1), 11.10.1–11.10.33. 10.1002/0471250953.bi1110s43 PMC4243306 25431634

[B31] Wang S. Sun J. Chen K. Ma P. Lei Q. Xing S. (2021). Perspectives of tumor-infiltrating lymphocyte treatment in solid tumors. BMC Med. 19 (1), 140. 10.1186/s12916-021-02006-4 34112147 PMC8194199

[B32] Wang Y. Navin N. E. (2015). Advances and applications of single-cell sequencing technologies. Mol. Cell 58 (4), 598–609. 10.1016/j.molcel.2015.05.005 26000845 PMC4441954

[B33] Warburton P. E. Sebra R. P. (2023). Long-read DNA sequencing: recent advances and remaining challenges. Annu. Rev. Genom Hum. Genet. 24, 109–132. 10.1146/annurev-genom-101722-103045 37075062

[B34] Wienert B. Wyman S. K. Richardson Christopher D. Yeh C. D. Akcakaya P. Porritt Michelle J. (2019). Unbiased detection of CRISPR off-targets *in vivo* using DISCOVER-Seq. Sci. (1979) 364 (6437), 286–289. 10.1126/science.aav9023 PMC6589096 31000663

[B35] Wu X. Kriz A. J. Sharp P. A. (2014). Target specificity of the CRISPR-Cas9 system. Quant. Biol. 2 (2), 59–70. 10.1007/s40484-014-0030-x 25722925 PMC4338555

[B36] Yu X. Abbas-Aghababazadeh F. Ann Chen Y. Fridley B. L. (2021). Statistical and bioinformatics analysis of data from bulk and single-cell RNA sequencing experiments. Methods Mol. Biol. 2194, 143–175. 10.1007/978-1-0716-0849-4_9 32926366 PMC7771369

[B37] Zeng Z. Chew H. Y. Cruz J. G. Leggatt G. R. Wells J. W. (2021). Investigating T cell immunity in cancer: achievements and prospects. Int. J. Mol. Sci. 22 (6), 2907. 10.3390/ijms22062907 33809369 PMC7999898

