# Supplementary material for: Assessing a single-cell multi-omic analytic platform to characterize ex vivo-engineered T-cell therapy products
Source: Front Bioeng Biotechnol. 2024 Aug 20;12:1417070. doi: 10.3389/fbioe.2024.1417070 (PMC11368872; doi:10.3389/fbioe.2024.1417070)
Supplement: Supplementary file 2 [file DataSheet1.docx]

***Supplementary Material***

**Assessing a single-cell multi-omic analytic platform to characterize ex vivo-engineered T cell therapy products**

**Maryam Moshref ^1*^, Jerry Lo^2^, Andrew McKay^3^, Julien Camperi^1^, Joseph Schroer^1^, Norikiyo Ueno^4^, Shu Wang^5^, Saurabh Gulati^5^, Somayeh Tarighat^1^, Steffen Durinck^2^, Ho-Young Lee^1^, and Dayue Chen^1*^**

^1^ Cell Therapy Engineering and Development, Genentech, 1 DNA Way, South San Francisco, CA 94080, USA

^2^Oncology Bioinformatics, Genentech, 1 DNA Way, South San Francisco, CA 04080, USA

^3^ Pharma Technical Development Bioinformatics, Genentech, 1 DNA Way, South San Francisco, CA 04080, USA

^4^Cell and Gene Therapy Business Unit, Mission Bio, 400 E Jamie Ct, South San Francisco, CA 94080, USA

^5^Bioinformatics Department, Mission Bio, 400 E Jamie Ct, South San Francisco, CA 94080, USA

*** Correspondence:**Maryam Moshref, [moshref.genentech@gmail.com](mailto:mmoshref@ucdavis.edu) and Dayue Chen, [chend66@gene.com](mailto:chend66@gene.com)

# Supplementary Material and Method

*Translocation assay by ddPCR*

Whole genomic DNA were isolated from edited and unedited cells following manufacturer’s instructions (Qiagen, #69504) and were then subjected to ddPCR translocation assay.

The percent of potential chromosomal translocations from CRISPR-mediated editing of three target sites (TRAC, TRBC1, and TRBC2) in engineered T Cells were measured on Bio-Rad’s QX200 ddPCR platform using six translocation assays: *TRAC-TRBC1, TRAC-TRBC2, TRBC1-TRBC2, TRBC2-TRBC1, TRBC2-TRAC,* and *TRBC2-TRBC1.* The target assays were labeled with FAM-fluorescent dye and the reference assay (RPP30) was labeled with HEX-fluorescent dye. Briefly, the translocation assay was performed by generating oil emulsion droplets containing the MasterMix, DNA of interest for amplification, and appropriate enzymes, which all acted as reaction

vessels for the digital droplet PCR. The abundance of each translocation was calculated relative to reference assay using the following equation shown below:

Translocation (% Ratio) = [Target(copies/uL)/Reference(copies/uL)] x 100. The primer and probe sequences are as follows:

TRAC forward, 5'-TGGGGCAAAGAGGGAAATGAG-3', TRAC reverse, 5' AGAACCTGGCCATTCCTGAAG-3'; TRAC-Probe, 5'-CATGTG CAAACGCCTTCAACAACAG-3'; TRBC1, 5'-CTGGGATGGTGACCCCAAAA-3'; TRBC1 reverse, 5'-GGCCACATAGAAAGGGGACC-3'; TRBC1, probe, 5'ACCATGAAGGAGAATTGGGCACCT-3'; TRBC2 forward, 5'-GGGGGATGGACAGACAATGG-3'; TRBC2 reverse, 5'- GCTGACCCTGTGAACCTTGA-3'; TRBC2-Probe: 5'-ATCCAGGTA GCGGACAAGACTAGAT-3'; RPP30 forward, 5'-TCAGCCATATTG TCCCCTAAACT-3'; RPP30 reverse, 5'-TGGTCTGTCCATGGCATC TT-3' and RPP30 probe, 5'-CTGTATGGACACAGTGCCTA-3'.

​​*T cell phenotype panel*

Transfected and non-transfected T cell samples were washed with FACS buffer being resuspended in FcR blocking solution and incubated for 10 minutes at 4°C. After incubation, cells were incubated with the antibody cocktail (Table S3 and Supplementary Figure 4) including anti-human CD8, CD27, CD45RO, CD45RA, CD3, CD95, CD62L, and CD197. The one isotype well was stained with Control Antibodies of the same fluorophores at the same dilutions. The plate was then incubated in the dark at 4 °C for 30 min. After incubation, cells were pelleted, washed twice with PBS, and gently resuspended in a prediluted Dead Viability Dye working solution. Afterward, cells were washed and re-suspended in the FACS buffer before proceeding to FACS acquisition. A conventional gating strategy was used to remove aggregates, and dead cells were excluded based on viability dye staining.

**Supplementary Tables**

| TRAC | TRBC1 |
| --- | --- |
| TRAC | TRBC2 |
| TRBC1 | TRCB2 |
| TRBC2 | TRBC1 |

**Supplementary Table1:** Editing 3 loci creates 6 possible translocations

| **Manufacturer** | **Barcode** | **Antibody** | **Clone** |
| --- | --- | --- | --- |
| BioLegend, TotalSeq-D | 152 | CD223 | 11C3C65 |
| BioLegend, TotalSeq-D | 176 | CD39 | A1 |
| BioLegend, TotalSeq-D | 89 | TIGIT | A15153G |
| BioLegend, TotalSeq-D | 1169 | CD279 | A17188B |
| BioLegend, TotalSeq-D | 147 | CD62L | DREG-56 |
| BioLegend, TotalSeq-D | 156 | CD95 | DX2 |
| BioLegend, TotalSeq-D | 169 | CD366 | F38-2E2 |
| BioLegend, TotalSeq-D | 148 | CD197 | G043H7 |
| BioLegend, TotalSeq-D | 63 | CD45RA | HI100 |
| BioLegend, TotalSeq-D | 154 | CD27 | O323 |
| BioLegend, TotalSeq-D | 72 | CD4 | RPA-T4 |
| BioLegend, TotalSeq-D | 46 | CD8a | SK1 |
| BioLegend, TotalSeq-D | 87 | CD45RO | UCHL1 |
| BioLegend, TotalSeq-D | 34 | CD3 | UCHT1 |

**Supplementary Table 2:** Oligo-tagged Antibodies used for Tapestri

| **Reagent** | **Manufacturer** | **Catalog Number** |
| --- | --- | --- |
| Mouse IgG1-APC-Cy7 | BioLegend | 400128 |
| Mouse IgG1- PerCP/Cy5.5, κ | BioLegend | 400150 |
| Brilliant Violet 421 Mouse IgG2a, κ | BioLegend | 400260 |
| PE Mouse IgG2b, κ | BioLegend | 400314 |
| Mouse IgG1-APC | BD Biosciences | 555751 |
| PE-Cy7 Mouse IgG1 κ | BD Biosciences | 557872 |
| BV605 Mouse IgG1, κ | BD Biosciences | 562652 |
| Alexa Fluor 700 Mouse IgG2a, κ | BD Biosciences | 557880 |
| CD8-APC-Cy7 | BioLegend | 300926 |
| CD27-PerCP-Cy5.5 | BioLegend | 302820 |
| CD45RO-BV421 | BioLegend | 304224 |
| CD45RA-PE | BioLegend | 304108 |
| CD3-APC | BD Biosciences | 340440 |
| CD95-PE-Cy7 | BD Biosciences | 561633 |
| CD62L-BV605 | BD Biosciences | 562720 |
| CD197(CCR7)-AF700 | BD Biosciences | 561143 |
| Human TruStain FcX | Biolegend | 422302 |

**Supplementary Table3:** Antibodies used for flow cytometric analyses

**Supplementary Figures:**

**A**
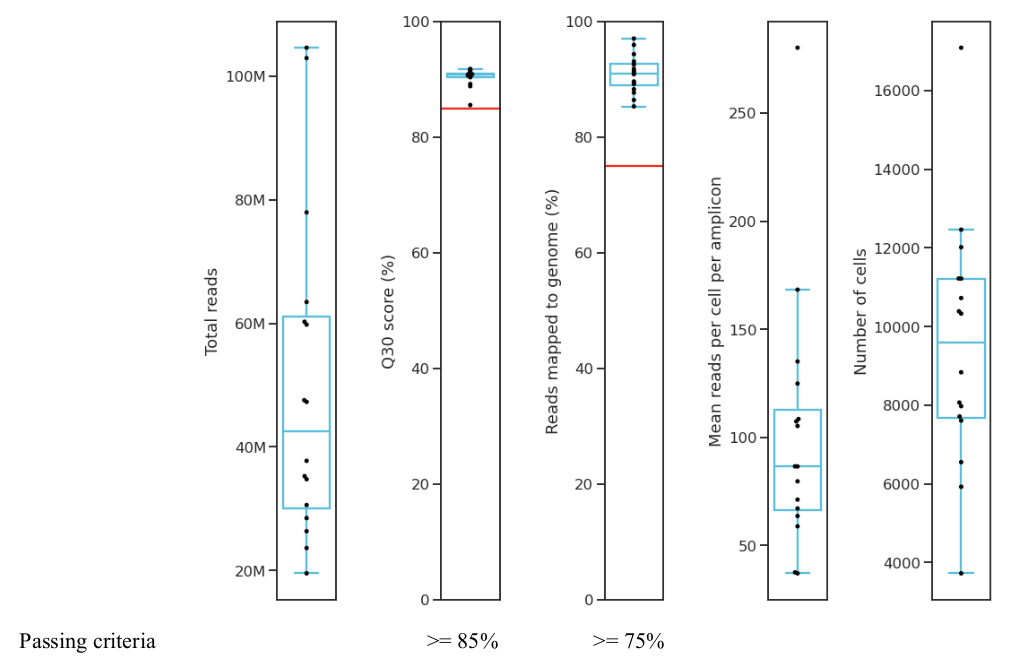


**B**
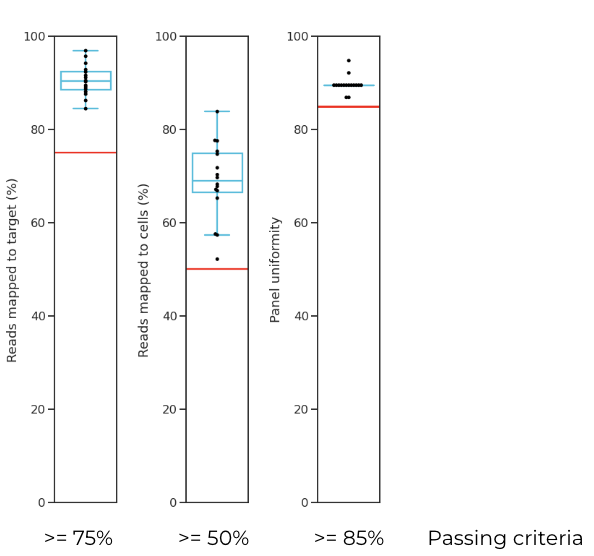


**Supplementary Figure 1:** Sequencing performance metrics (A) and Panel performance metrics (B) are within QC specs.

**A**

**
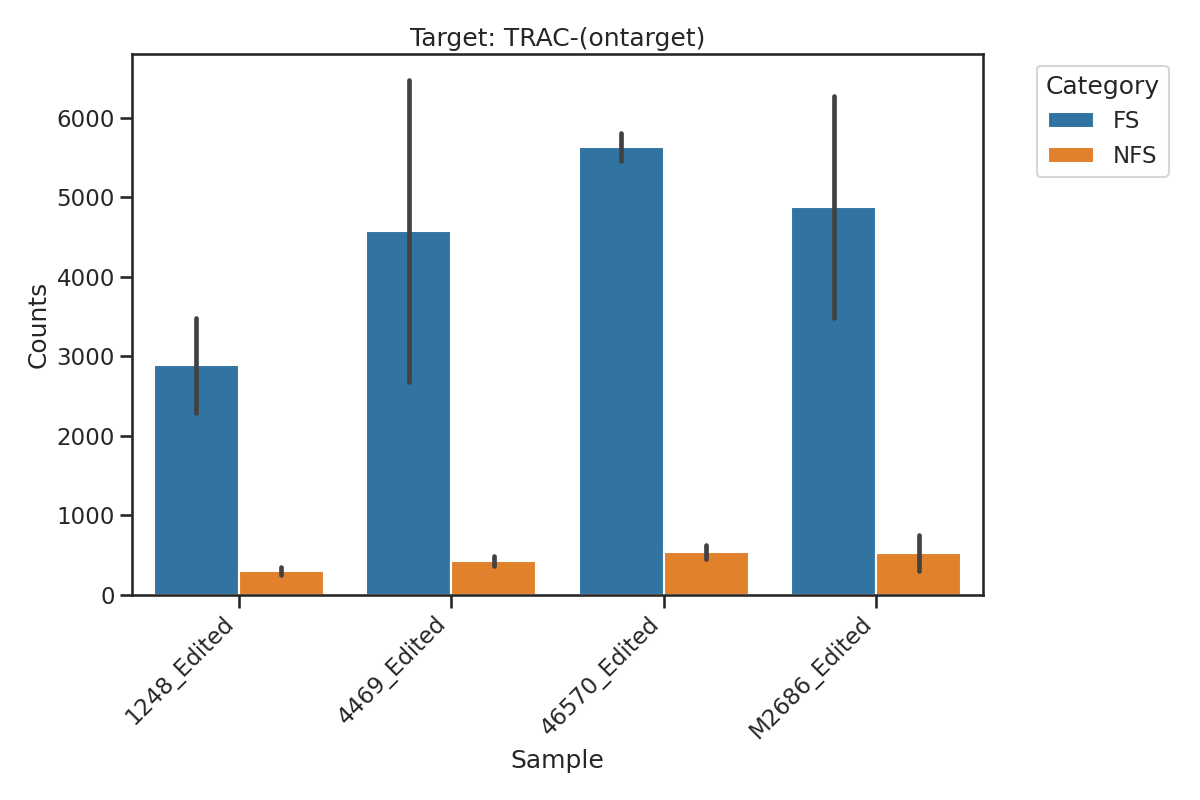
**

**B**

**
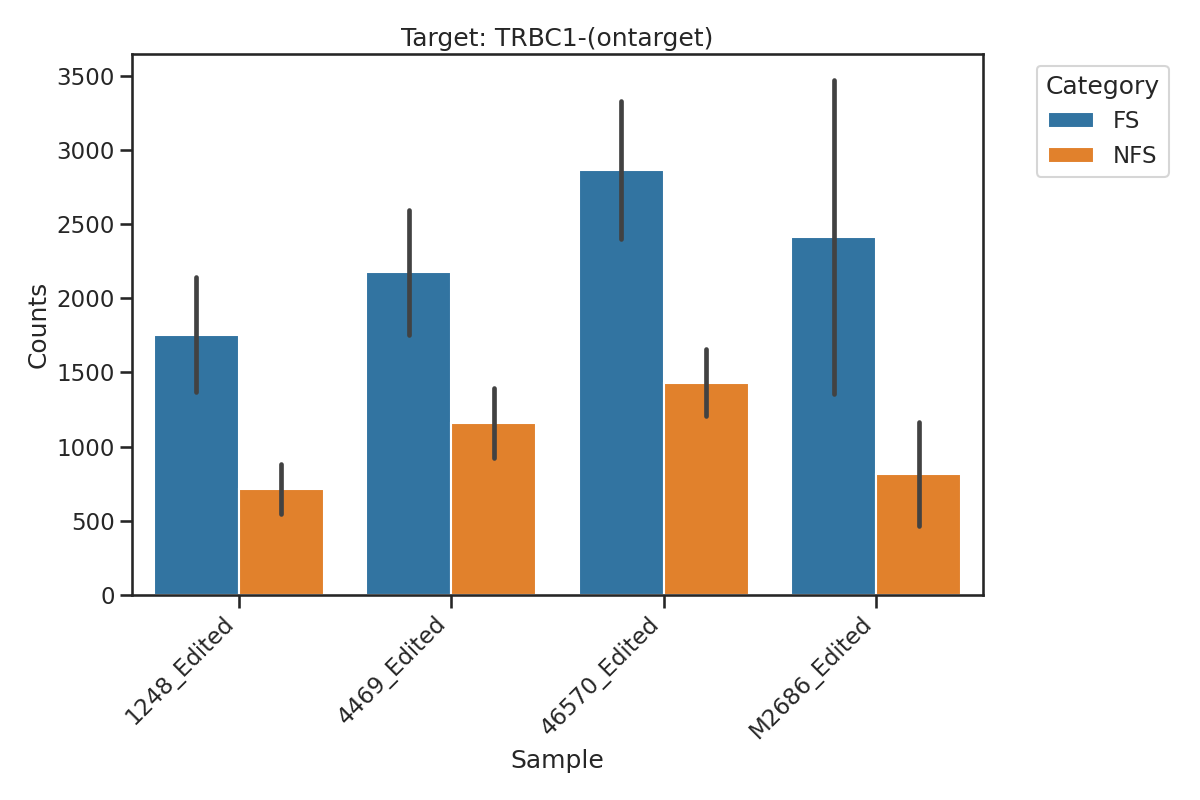
**

**C**

**
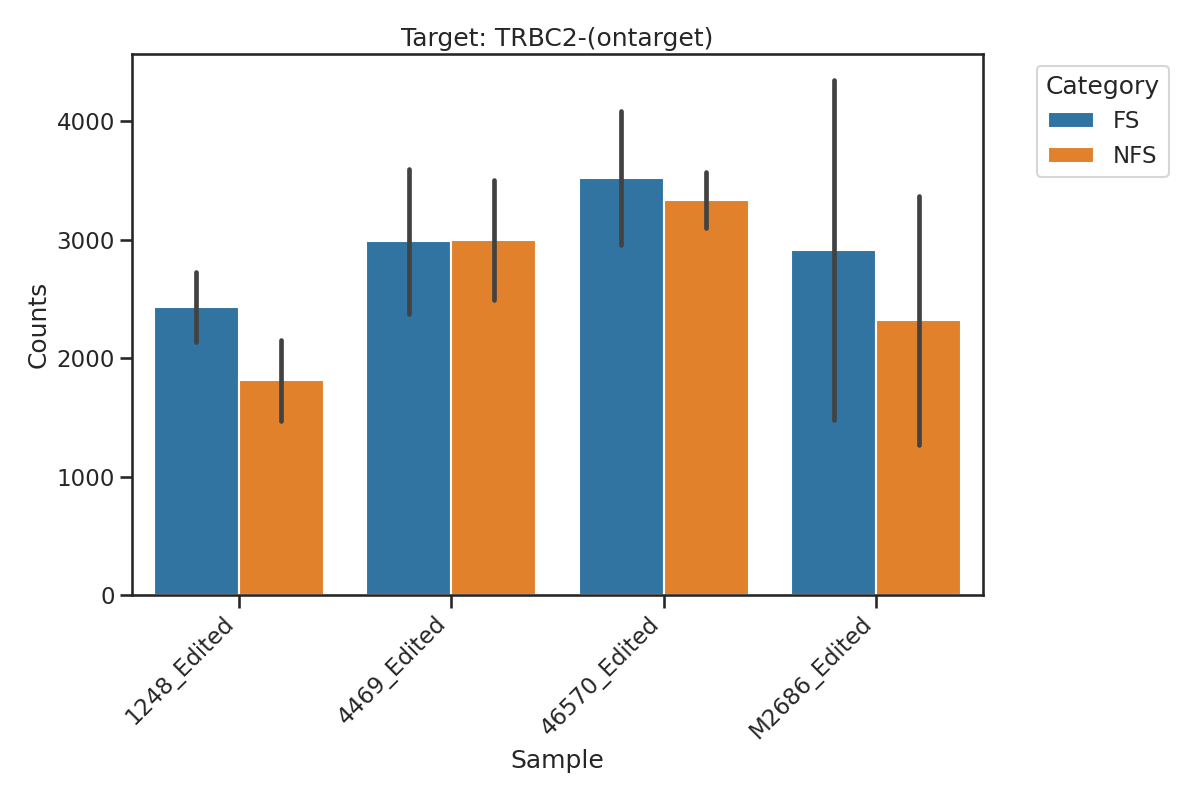
**

**Supplementary Figure 2:** Frame shift analysis of A) TRAC locus, B) TRBC1 locus and C) TRBC2 confirmed that the 3 loci were disturbed by CRISPER_Cas9. Error bars represent the standard deviation of the technical replicates.

**
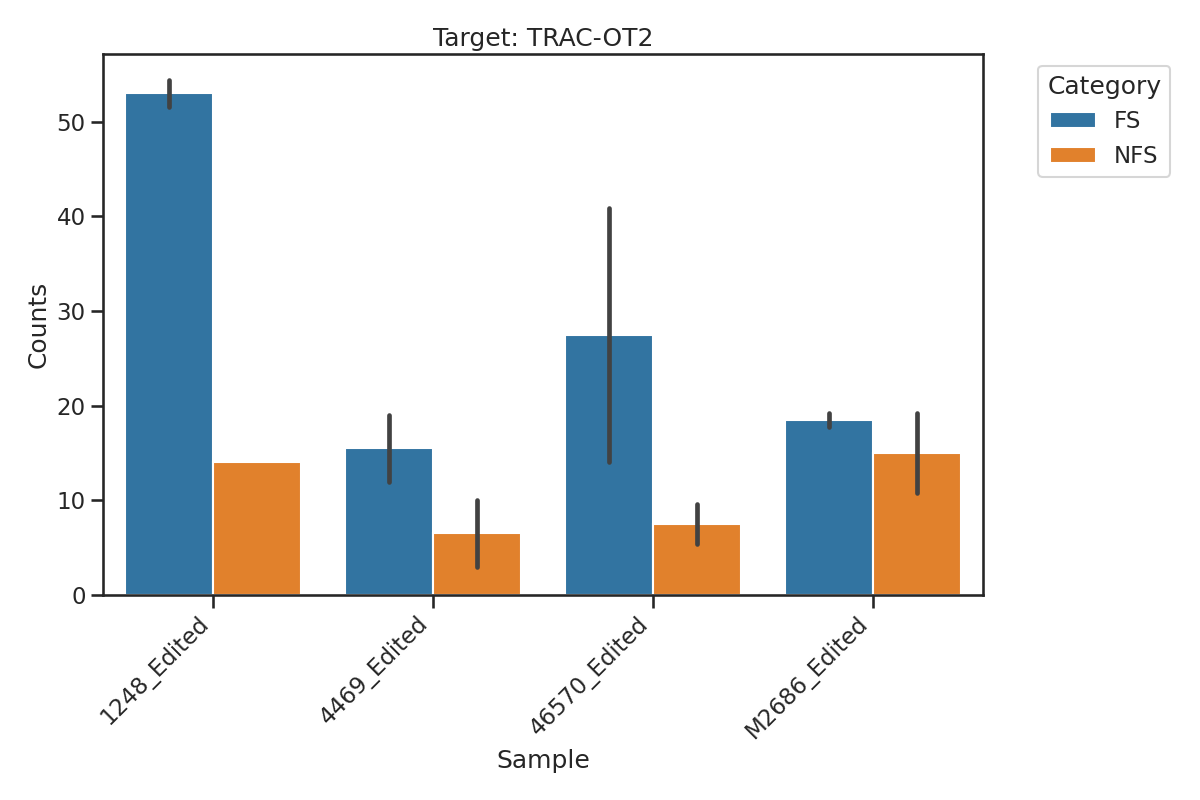
**

**Supplementary Figure 3:** Frameshift analysis of TRAC-OT2 suggest that TRAC-OT2 is an off target.

**
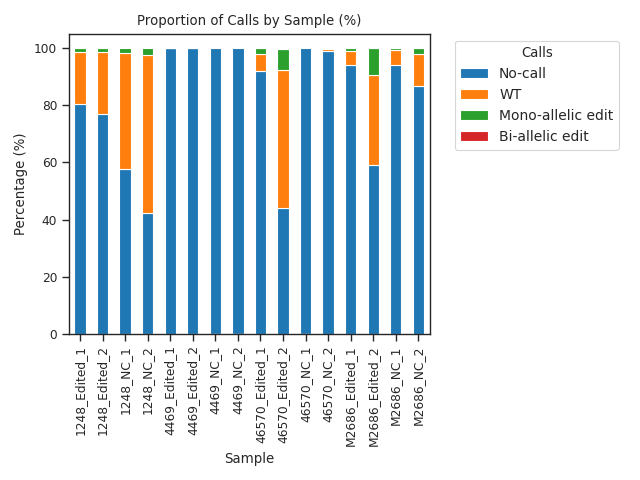
**

**Supplementary Figure 4:** TRAC-OT10 genotype calls are dominated by No-calls due to insufficient read depth, resulting in this off-target locus being low performing.

**
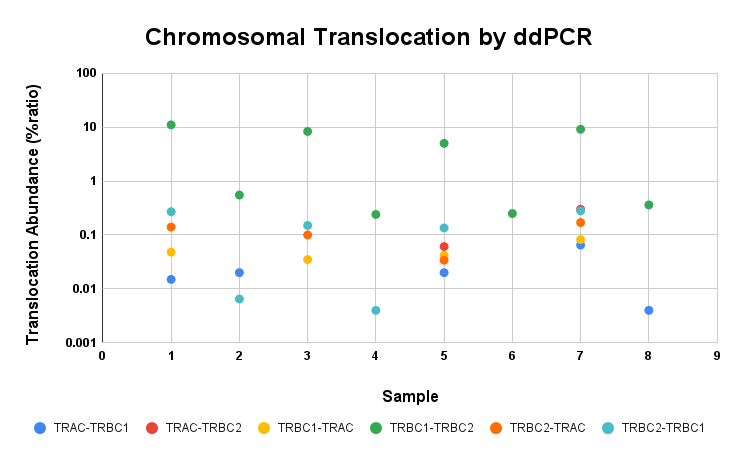
**

**Supplementary Figure 5:** Translocation abundance among different donors by bulk analysis (ddPCR)

**
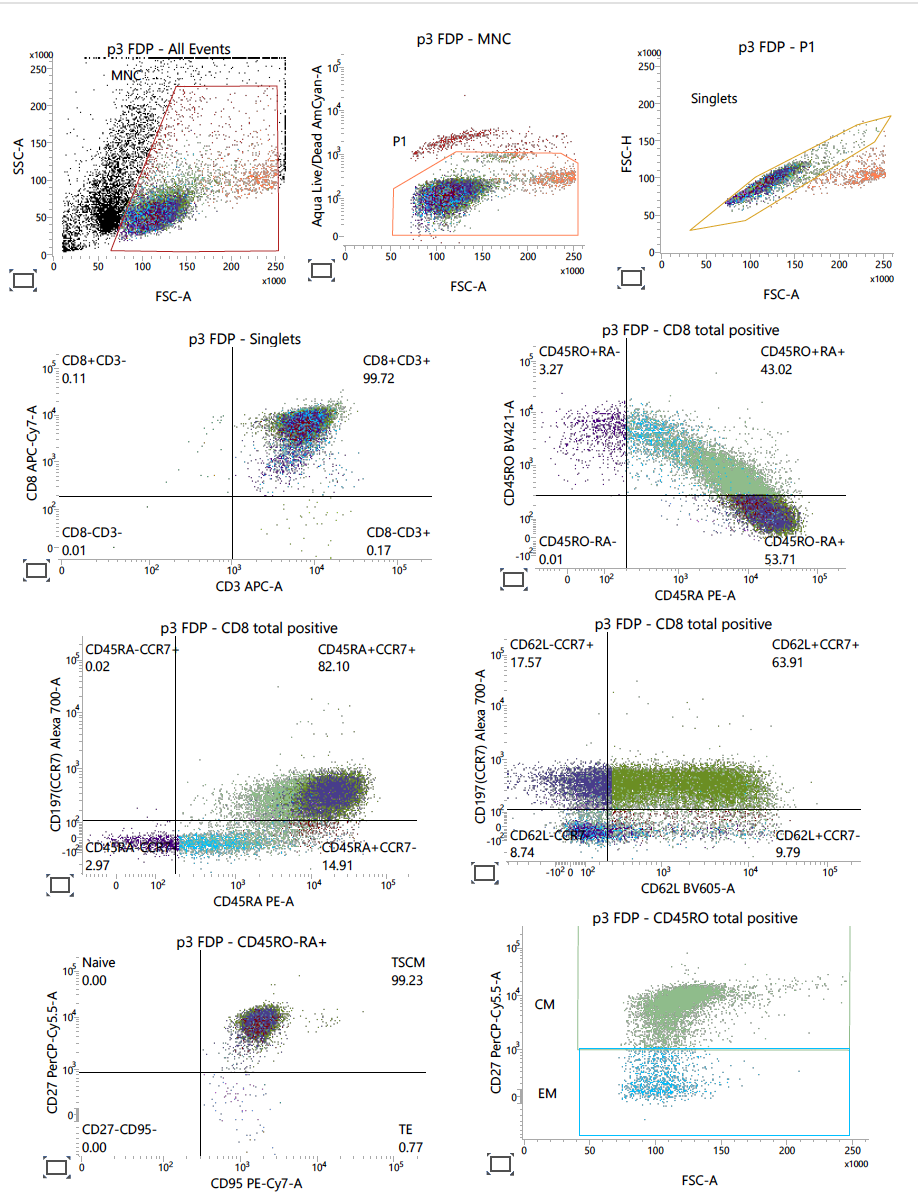
**

**
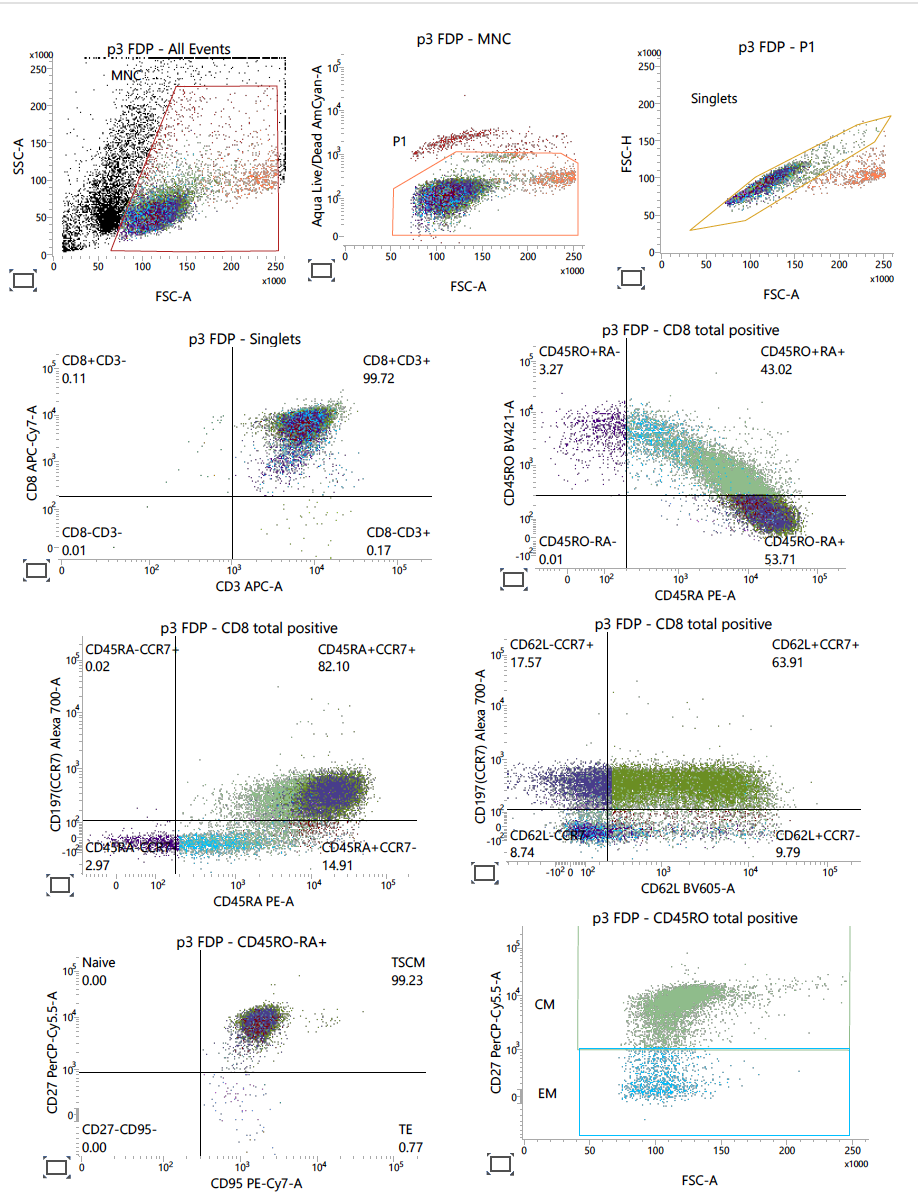
Supplementary Figure 6:** Phenotype panel of edited (top) and unedited (bottom) T cells (Donor 1) by flow-cytometry.

**
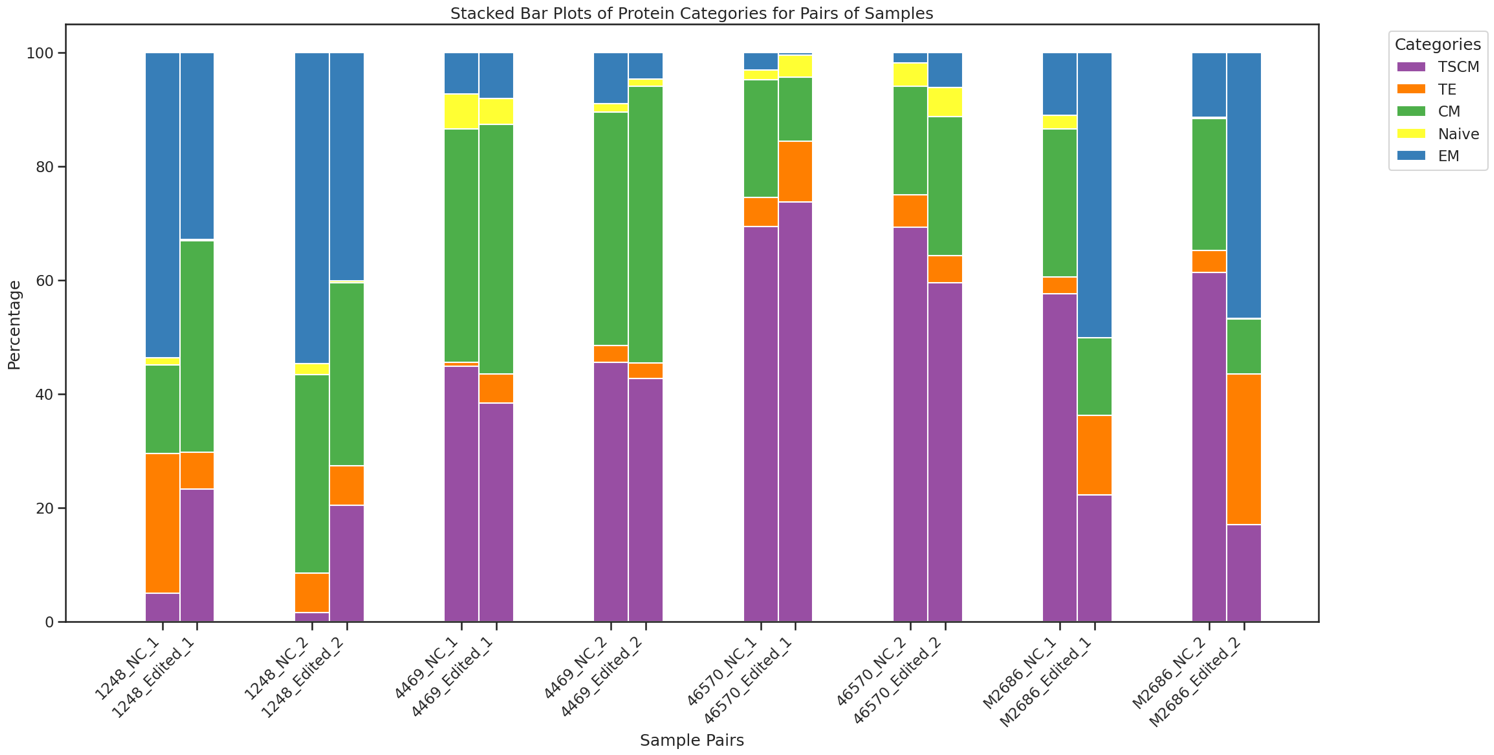
**

**Supplementary Figure 7:** Proportion of CD8 T cell subsets for cells with triple wildtype genotypes, paired by non-edited and edited technical replicates.
